# Integrative genomics analysis of chromosome 5p gain in cervical cancer reveals target over-expressed genes, including *Drosha*

**DOI:** 10.1186/1476-4598-7-58

**Published:** 2008-06-17

**Authors:** Luigi Scotto, Gopeshwar Narayan, Subhadra V Nandula, Shivakumar Subramaniyam, Andreas M Kaufmann, Jason D Wright, Bhavana Pothuri, Mahesh Mansukhani, Achim Schneider, Hugo Arias-Pulido, Vundavalli V Murty

**Affiliations:** 1 Department of Pathology, Columbia University Medical Center, New York, New York, USA; 2 Department of Molecular & Human Genetics, Banaras Hindu University, Varanasi, India; 3 Department of Gynecology, Charité Universitätsmedizin Berlin, Hindenburgdamm 30, Berlin, Germany; 4 Gynecologic Oncology, Columbia University Medical Center, New York, New York, USA; 5 Department of Gyenecologic Oncology, New York University Medical Center, New York, New York, USA; 6 Division of Hematology/Oncology, The University of New Mexico Cancer Center, 900 Camino de Salud NE, Albuquerque, New Mexico, USA; 7 Department of Tumor Molecular Biology, Instituto Nacional de Cancerología, Bogotá, Colombia; 8 Institute for Cancer Genetics, Columbia University Medical Center, New York, New York, USA; 9 Irving Cancer Research Center, Room 605, Columbia University Medical Center, 1130 St. Nicholas Ave, New York, New York 10032, USA

## Abstract

**Background:**

Copy number gains and amplifications are characteristic feature of cervical cancer (CC) genomes for which the underlying mechanisms are unclear. These changes may possess oncogenic properties by deregulating tumor-related genes. Gain of short arm of chromosome 5 (5p) is the most frequent karyotypic change in CC.

**Methods:**

To examine the role of 5p gain, we performed a combination of single nucleotide polymorphism (SNP) array, fluorescence in situ hybridization (FISH), and gene expression analyses on invasive cancer and in various stages of CC progression.

**Results:**

The SNP and FISH analyses revealed copy number increase (CNI) of 5p in 63% of invasive CC, which arises at later stages of precancerous lesions in CC development. We integrated chromosome 5 genomic copy number and gene expression data to identify key target over expressed genes as a consequence of 5p gain. One of the candidates identified was Drosha (*RNASEN*), a gene that is required in the first step of microRNA (miRNA) processing in the nucleus. Other 5p genes identified as targets of CNI play a role in DNA repair and cell cycle regulation (*BASP1*, *TARS*, *PAIP1*, *BRD9*, *RAD1*, *SKP2*, and *POLS*), signal transduction (*OSMR*), and mitochondrial oxidative phosphorylation (*NNT*, *SDHA*, and *NDUFS6*), suggesting that disruption of pathways involving these genes may contribute to CC progression.

**Conclusion:**

Taken together, we demonstrate the power of integrating genomics data with expression data in deciphering tumor-related targets of CNI. Identification of 5p gene targets in CC denotes an important step towards biomarker development and forms a framework for testing as molecular therapeutic targets.

## Background

The short arm of chromosome 5 (5p) frequently undergoes nonrandom changes in cervical cancer (CC) by exhibiting both copy number increase and deletions. Gain of 5p due to frequent appearance of isochromosome 5p in squamous cell carcinoma has been documented by karyotypic and chromosomal comparative genomic hybridization analyses [1-4]. Paradoxically, 5p also exhibits frequent loss of heterozygosity, which occurs early in the development of CC [5,6]. These findings suggest the presence of important proliferation-regulating genes on chromosome 5p involved in malignant progression of cervical epithelium.

Despite the successful use of pap-smear screening programs in early detection and treatment of CC, this tumor remains a major cause of cancer deaths in women world-wide [7]. CC progresses by distinct morphological changes from normal epithelium to carcinoma through low-grade squamous intraepithelial lesions (LSIL) and high-grade SILs (HSIL). Currently, no biological or genetic markers are available to predict which precancerous lesions progress to invasive CC. Although infection of high-risk human papillomavirus (HPV) is recognized as an essential initiating event in cervical tumorigenesis, this alone is not sufficient for the progression to invasive cancer [8]. In spite of the recent progress in molecular aspects of CC, the genetic basis of progression of precursor SILs to invasive cancer in the multi-step progression of CC remains poorly understood [9]. Therefore identification of other "genetic hits" in CC is important in understanding its biology.

Chromosomal gain and amplification is a common cellular mechanism of gene activation in tumorigenesis [10]. The aim of the present study was to examine the contribution of chromosome 5 copy number alterations (CNA) in CC tumorigenesis and identify copy number driven gene expression changes. We performed single nucleotide polymorphism (SNP) array and fluorescence in situ hybridization (FISH) analysis on invasive cancer and identified 5p CNI in a high frequency of primary tumors and cell lines. To unravel the consequence of 5p CNI on transcription, we utilized Affymetrix U133A gene expression array and identified a number of over expressed genes on 5p, which include *RNASEN*, *POLS*, *OSMR*, and *RAD1 *genes. These data, thus, suggest that transcriptional activation of multiple genes on 5p plays a role as driver genes in the progression of CC.

## Methods

### Tumor specimens and cervical cancer cell lines

A total of 219 specimens were utilized in the present study in various investigations. These include 9 cell lines, 148 primary tumors, 42 pap smears, and 20 normal cervical tissues. The cell lines (HT-3, ME-180, CaSki, MS751, C-4I, C-33A, SW756, HeLa, and SiHa) were obtained from American Type Culture Collection (ATCC, Manassas, VA) and grown in tissue culture as per the supplier's specifications. Twenty age-matched normal cervical tissues from hysterectomy specimens obtained from Columbia University Medical Center (CUMC), New York, were used as controls after enrichment for epithelial cells by microdissection. Cytologic specimens were collected using the ThinPrep Test Kit (Cytc Corporation, Marlborough, MA). After visualization of the cervical os the ectocervix was sampled with a spatula and endocervical cells obtained with a brush rotated three hundred sixty degrees. Exfoliated cells were immediately placed in PreservCyt Solution (Cytc Corporation, Marlborough, MA) for routine processing by a cytopathologist. Pap smears were collected from normal and precancerous lesions by simultaneous preparation of slides from the same spatula for both cytology and FISH. FISH slides were immediately fixed in 3:1 methanol and acetic acid, and stored at 4°C until hybridization. A total of 42 pap smears with the diagnosis rendered by a cytopathologist as normal/squamous metaplasia/ASCUS (N = 10), LSIL (N = 13) or HSIL (N = 19) obtained from CUMC were used for FISH analysis. The diagnosis of all HSILs was also confirmed by a biopsy. Of the 148 primary tumors, 93 were obtained as frozen tissues and 55 specimens as formalin-fixed paraffin-embedded tissues. All primary invasive cancer specimens were obtained from patients evaluated at CUMC, Instituto Nacional de Cancerologia (Bogota, Colombia) [11], and the Department of Gynecology of Campus Benjamin Franklin, Charité-Universitätsmedizin Berlin (Germany) with appropriate informed consent and approval of protocols by institutional review boards. All primary tumors were diagnosed as squamous cell carcinoma (SCC) except five that were diagnosed as adenocarcinoma (AC). Clinical information such as age, stage and size of the tumor, follow-up data after initial diagnosis and treatment was collected from the review of institutional medical records. Tissues were frozen at -80°C immediately after resection and were embedded with tissue freeze medium (OTC) before microdissection. All primary tumor specimens were determined to contain at least 60% tumor by examination of hematoxylin and eosin (H&E) staining of adjacent sections. High molecular weight DNA and total RNA from tumor, normal tissues, and cell lines was isolated by standard methods. The integrity of all RNA preparations was tested by running formaldehyde gels and samples that showed evidence of degradation were excluded from the study.

### Microarray analysis

The Affymetrix 250 K *Nsp*I SNP chip was utilized for copy number analysis as per the manufacturer's protocol. Briefly, 250 ng of genomic DNA was digested with *Nsp*I, generic linkers were added followed by PCR amplification, end-labeling, and fragmentation following standard protocols. Hybridization, washing, acquisition of raw data using GeneChip Operating Software (GCOS), and generation of .CEL files was performed by the Affymetrix Core facility at our institute. We utilized 79 CC cases (9 cell lines and 70 primary tumors enriched for tumor cells by microdissection) and 7 microdissected normal cervical squamous epithelial samples as controls to serve as reference for copy number analysis. SNP data of test samples and normal cervical epithelial specimens were loaded to dChip to calculate signal intensity values using the perfect match/mismatch (PM/MM) difference model followed by normalization of signals within chip and between chips using model-based expression [12,13]. DNA copy number gains were obtained as determined by dChip using analysis of signal intensity values based on the Hidden Markov Model. Arrays with > 93% call rates were included in the analysis as per Affymetrix manual. Copy number data was obtained for chromosome 5 using CytoBand information files from the dChip website [14]. Both the raw copy number and log_2 _ratio (Signal/mean signal of normal samples at each SNP) were computed to estimate copy number changes in chromosome view. Copy numbers < 1.5 were considered as deletion, 2.5 or more as gain in the raw copy number view. All the original data files were submitted to Gene Expression Omnibus (GEO Accession number: GSE10092).

We utilized Affymetrix U133A oligonucleotide microarray (Santa Clara, CA) containing 14,500 probe sets for gene expression analysis. RNA isolated from 30 CC cases (21 primary tumors enriched for tumor cells by microdissection and 9 cell lines) and 20 microdisssected normal cervical squamous epithelial cells were utilized for expression studies. Biotinylated cRNA preparation and hybridization of arrays was performed by the standard protocols supplied by the manufacturer. Arrays were subsequently developed and scanned to obtain quantitative gene expression levels. Expression values for the genes were determined using the Affymetrix GeneChip Operating Software (GCOS) and the Global Scaling option, which allows a number of experiments to be normalized to one target intensity to account for the differences in global chip intensity. The .CEL files obtained from the GCOS software were processed and normalized by dChip algorithm as described above. An average percent present call of 54% was obtained among all samples, which is expected for high quality RNA as per the manufacturer. Arrays were normalized at PM/MM probe level and a median intensity array from normal as the baseline array using invariant set normalization [12,13]. Followed by normalization, model based expression values were calculated using PM/MM data view to fit the model for all probe sets. All original data files were deposited to GEO (Accession number: GSE9750). To obtain a list of differentially expressed gene signatures, we compared all normal with all tumor samples using the criteria of 1.75-fold change between the group means at 90% confidence interval and a significance level of P < 0.05. All negative expression values for each probe set were truncated to 1 before calculating fold changes and < 10% of samples with present call in each group were excluded. A list of differentially expressed genes identified on chromosome 5 was used in all subsequent supervised analyses using the same criteria between various groups to obtain relevant gene signatures.

### Fluorescence in situ hybridization (FISH) and HPV typing

FISH was performed by standard methods on frozen tissue sections fixed in 3:1 methanol: acetic acid, tissue microarrays prepared from paraffin embedded tissues, and on pap smears fixed in 3:1 methanol: acetic acid. A dual color locus specific probe set containing spectrum orange labeled EGR1 (map to 5q31) and spectrum green labeled D5S23/D5S721 (map to 5p15.2) was obtained from Vysis (Downers Grove, IL). Hybridization signals on 100–500 interphase cells on DAPI counterstained slides were scored on Nikon Eclipse epi-fluorescence microscope equipped with Applied Imaging CytoVision software (San Jose, CA). Scoring of FISH signals on frozen and paraffin-embedded tissue sections was restricted to tumor cells based on the identification of areas of tumor on adjacent H&E sections by the pathologist (MM). FISH signal scoring on Pap smear slides was restricted to large and atypical epithelial cells. Presence of signals suggestive of gain in at least 3% cells was considered positive and the results correlated with parallel cytomorphologic findings. Human papillomavirus types were identified as described earlier [15].

## Results

### Identification of 5p gain as the most frequent genomic alteration in invasive CC

**Figure 1:**
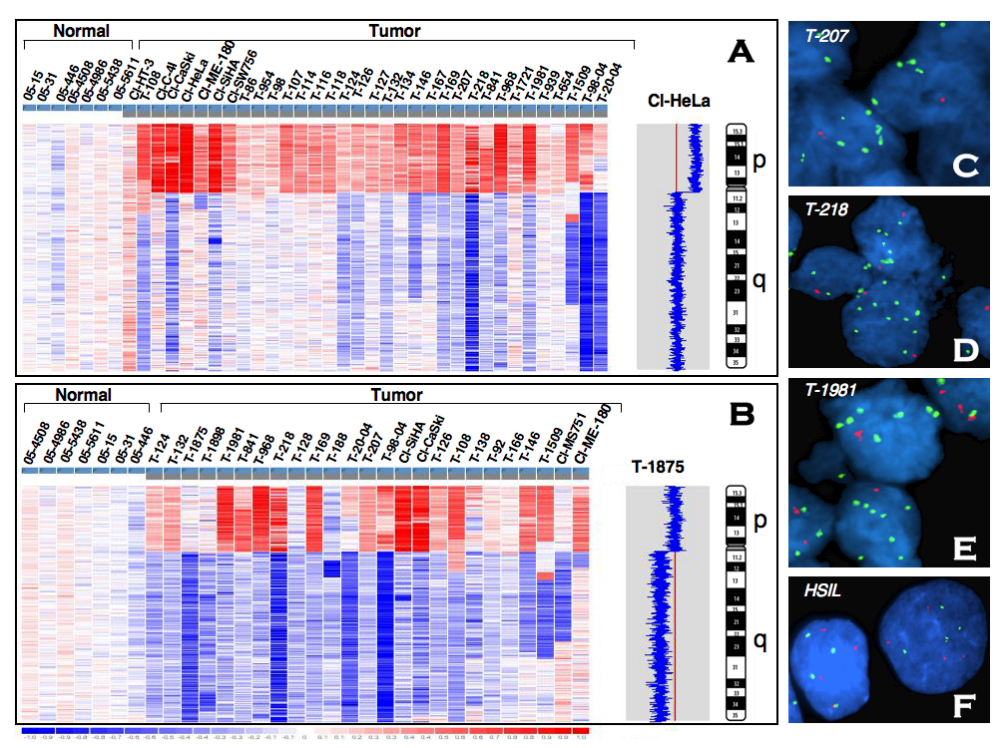
I**dentification of chromosome 5p genomic alterations in cervical cancer.A-B**. Copy number alterations in log2 ratio of chromosome 5 identified by 250 K *Nsp*I SNP array. Each vertical column represents a sample with genomic regions representing from pter (top) to qter (bottom). Prefix "T" indicates primary tumor; "CL" indicates cell line. The blue-red scale bar (-1 to +1) at the bottom represents the copy number changes relative to mean across the samples. The intensities of blue and red indicate relative decrease and increase in copy numbers, respectively. G-banded ideogram of chromosome 5 is shown on the extreme *right *in each panel. **A**. Identification of 5p gain as the most frequent genomic alteration in CC. All tumors that exhibited 5p gain are shown. Inferred copy number view of HeLa cell line showing 5p gain from normal (2N) (red line) is shown on *right*. **B**. 5q deletions in CC. All tumors that exhibited 5q deletions arranged from largest to smallest deletion are shown. Inferred copy number view of T-1875 showing 5q deletion from normal (2N) (red line) is shown on *right*. **C-F**. Fluorescence in situ hybridization (FISH) identification of 5p gains in invasive cancer (**C-E**) and high-grade cervical intra-epithelial lesion (HSIL) (**F**). Green signals represent 5p15.2 probe and Red signals represent probe mapping to 5q31 region used as control. Panels C-D represent invasive CC cases with increased copies of 5p (green) compared to 5q (red). Note the concordance of SNP copy number changes (panel A) and increased copies of 5p by FISH (panels C-E) for tumors T-207, T-218, and T-1981, respectively. Panel F showing 4 copies of chromosome 5p and 5q (tetrasomy 5) on a pap smear from HSIL.

Affymetrix 250 K *Nsp*I SNP array analysis was performed on a panel of 79 CC cases (70 primary tumors and 9 cell lines) to identify genome-wide copy number alterations (CNA) (unpublished data). The dataset of chromosome 5 CNA from this analysis was utilized in the present study. CNA of chromosome 5 was found in 42 (53.2%) CC cases. Of these, 5p exhibited copy number gains in 34 (43%) cases while no detectable copy number losses were found on this chromosomal arm (Figure 1A). Gain of 5p was the most commonly affected regions in the CC genome (see Additional file 1). On the other hand, gain of long arm of chromosome 5 (5q) was rare with only 3 (3.8%) tumors showing CNI. However, copy number losses on 5q were found in 25 (31.6%) tumors. Of these, 17 had concurrent 5p gains and 5q losses, while the remaining 8 only showed 5q deletion (Figure 1B). Among the tumors that exhibited 5p CNI the entire 5p was gained and no minimal region of duplication or amplification could be delineated. Similarly, deletions on 5q span large regions often spanning the entire chromosomal arm and no consensus minimal deletion could be identified (Figure 1B). This data demonstrate that the chromosome 5p is a frequent target of CNI in CC, while accompanying deletions on 5q were found less frequently. To identify the clinical significance, we evaluated the association of chromosome 5 CNA with pathologic features such as histology, age, stage and size of the tumor, treatment outcome, and HPV type by univariate analyses and found no significant associations. These data thus suggest that chromosome 5 CNA is a critical genetic alteration that may occur early in the development of CC.

### FISH validation of 5p gain in CC

To validate the 5p gain identified by SNP array, we performed FISH analysis using a cocktail of two probes containing spectrum green-labeled 5p15.2 locus and spectrum orange-labeled 5q31 region on 101 CC cases. These include an independent panel of 55 tumors on a paraffin-embedded tissue microarray and an additional 46 tumors as frozen sections or pap smears. The latter include 23 tumors studied by SNP array (see Additional file 2). A total of 64 (63%) tumors showed an evidence for increased copies (3 or more) of 5p (Figure 1C–E). An average of 4.4 copies (range 3–11) of 5p15 signals were found among the 64 cases that exhibited gain, while only 2.6 copies (range: 1–8) of the 5q31 region were present (Figure 1C–E). These data, thus, suggest that the 5p CNI is independent of ploidy of the tumor and support the SNP data showing the gain of 5p and associated loss of 5q.

All the tumors that exhibited 5p gain by SNP array also showed gain by FISH. For example, the tumors T-207, T-218, and T-1981 showed simultaneous high copy numbers of 5p and loss of 5q by SNP array analysis. The FISH results on the same tumors are in complete agreement with the SNP data (Figure 1). These results, thus, validate the SNP data and establish that 5p CNI as the most frequent genetic alteration in CC.

### Chromosome 5p gain is a late genetic event in CC progression

CC progresses through distinct morphological changes during the transition from normal epithelium to carcinoma through low- and high-grade SILs. To identify the earliest stage in CC development in which the 5p CNI occur, we used a FISH assay on 42 consecutively ascertained pap smears simultaneously diagnosed by cytology as normal, squamous metaplasia or with atypical cells of undetermined significance (ASCUS) (N = 10), LSIL (N = 14) and HSIL (N = 19). Five of 19 (26.3%) HSILs showed four or more copies (range 4–7) of 5p (Figure 1F). Of these, three HSILs exhibited tetrasomy 5 while 2 others showed evidence of 5p gain (5–7 copies vs. 3–4 copies of 5q) (Figure 1F). No evidence of gain of 5p was found in any specimens diagnosed as LSIL, normal, squamous metaplasia or ASCUS. Thus, these data suggest that 5p gain is a relatively late event in the progression of CC.

The biological behavior of HSILs varies where only a small proportion progresses to invasive cancer if left untreated [16-18]. Cytologic characterization alone doesn't permit the identification of HSILs at risk for progression from those that regress or persist. Because of this, all HSILs are currently treated by surgical excision or with an ablative therapy. Identification of genetic signatures defining the subset of high-risk HSILs could alter the treatment strategies. Chromosome 5p gain may serve as such a genetic marker in predicting the progression of HSILs.

### Identification of transcriptional targets of 5p gain, including Drosha, in CC

We have shown 5p CNI as the most frequent genomic alteration in CC by combined SNP (see Additional file 1) and FISH analyses. We hypothesize that the increased 5p dosage may result in deregulation of genes that may confer oncogenic properties to its host cell. To identify such transcriptional targets on 5p, we utilized gene expression profiling data on Affymetrix U133A array analysis of 20 normal squamous epithelial samples (age range, 27–64 yr; Mean ± SD, 46.9 ± 7.6) and 30 CC cases (21 primary tumors; age range 28–70 yr; Mean ± SD, 48.3 ± 11.3; and 9 cell lines). Initial identification of differentially expressed gene signatures on chromosome 5 in CC was obtained by comparison of all probe sets on chromosome 5 present in U133A array between tumors and normal that exhibit significant (P < 0.05) differences using the criteria described in materials and methods. This algorithm identified 122 non-redundant probe sets with significant differences in expression levels in tumors compared to normal. This unique CC chromosome 5 gene signature, which distinguishes normal from tumor, includes 26 probe sets with down-regulated expression and 96 probe sets with increased expression (see Additional file 3). We anticipate that this differentially expressed gene data set will be useful in identifying target genes of CNI of 5p and loss of 5q in CC. Therefore, we focused our attention on this gene dataset in all subsequent supervised analyses of gene expression.

**Figure 2:**
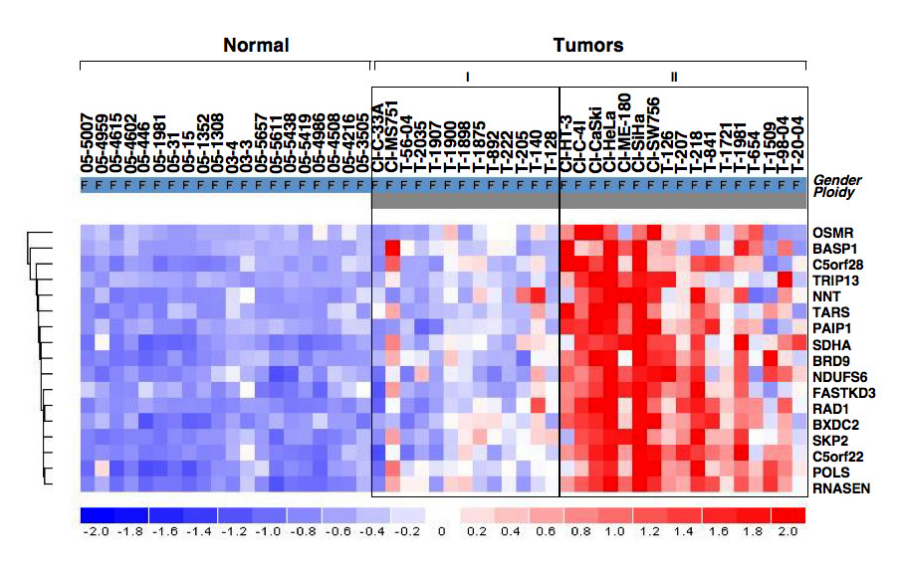
**Supervised analysis of over expressed genes identified as a consequence of gain of chromosome 5q in cervical cancer**. Significantly differentially expressed genes were identified by filtering all the over expressed genes on chromosome 5p between tumor that showed gain of 5p and tumors with out 5p gain. In the matrix, each row represents the gene expression relative to group mean and each column represents a sample (shown on Top). T, represents primary tumor; CL, represents cell line. The dendrogram on left shows unsupervised clustering of genes differentially expressed between tumors with and without gain. The names of genes are shown on right. The scale bar (-2 to +2) on the bottom represents the level of expression with intensities of blue represents decrease and red for increase in expression. The groups within tumors shown at top represent no gain of chromosome 5p (I) and 5p gain (II).

To further identify over expressed genes on 5p, we then filtered all over expressed probe sets on chromosome 5 against all 5p probes on U133A array and this analysis showed enrichment for over expressed genes by identifying 26 genes (*LOC728411*, *PDCD6*, *RAI14*, *SLC12A7*, *DAP*, *Hs.561432*, *MYO10*, *OSMR*, *BASP1*, *C5orf28*, *TRIP13*, *NNT*, *TARS*, *PAIP1*, *CCT5*, *SDHA*, *NDUFS6*, *BRD9*, *KIAA0947*, *FASTKD3*, *RAD1*, *BXDC2*, *SKP2*, *C5orf22*, *POLS*, and *RNASEN*) (see Additional file 4). We then asked whether the over expression of these 26 genes in CC compared to normal squamous epithelia is due to a generalized tumor-related transcriptional deregulation or is a consequence of 5p CNI. To examine this, we filtered the 26 over expressed gene data set by comparison between tumors exhibited 5p gain (N = 17) and tumors with disomy 5p (N = 13) by SNP analysis. This supervised analysis identified 17 differentially over expressed genes (*OSMR*, *BASP1*, *C5orf28*, *TRIP13*, *NNT*, *TARS*, *PAIP1*, *SDHA*, *NDUFS6*, *BRD9*, *FASTKD3*, *RAD1*, *BXDC2*, *SKP2*, *C5orf22*, *POLS*, and *RNASEN*) only in tumors exhibiting 5p CNI (Figure 2). Therefore, these 17 genes represent copy number driven target over expressed genes that likely provide growth advantage and invasion conferred by chromosome 5p gain.

Although a similar type of analysis identified down-regulated gene signature on 5q in invasive CC, no specific signature associated with 5q deletion could be identified (see Additional file 5). Analysis performed to identify the down regulated genes on 5q using all probes on chromosome 5q on U133A array showed a total of 17 down regulated genes (*EGR1*, *PITX1*, *MAST4*, *GALNT2*, *ATP10B*, *DUSP1*, *HBEGF*, *RMND5B*, *HMGCR*, *CAST*, *CLTB*, *GX3*, *SPINK5*, *LOC653314*, *CXCL14*, *ISL1*, and *PIK3R1*) in CC compared to normal cervical epithelium. Thus, these data suggest that the 5p gain is a critical genetic change in CC and the genes identified as a consequence of 5p gain may be important in its tumorigenesis. These data further suggest that the 5q loss may have little consequence to CC biology and may represent a by-stander genetic alteration associated with 5p gain.

## Discussion

We provide multiple levels of evidence to support that genomic gain of chromosome 5p is an important genetic target in CC development. First, our SNP analysis identified 5p gain as the most frequent genetic alteration in invasive CC (see Additional file 1). By FISH we confirmed this finding using an independent cohort of CC specimens and seen only in high-grade SILs. Several previous studies have identified recurrent gain of 5p in many types of human cancers [19], including CC [1-4,20-22]. Gain of 5p also appears to arise at latter passages in HPV-immortalized cervical keratinocytes and its acquisition confers the ability to invade collagen in tissue culture [23]. This close recapitulation of 5p gain in latter stages of an in vitro model and in the clinical specimens from CC patients provide a strong evidence that this change occurs late in the development and may play role in invasion.

**Figure 3:**
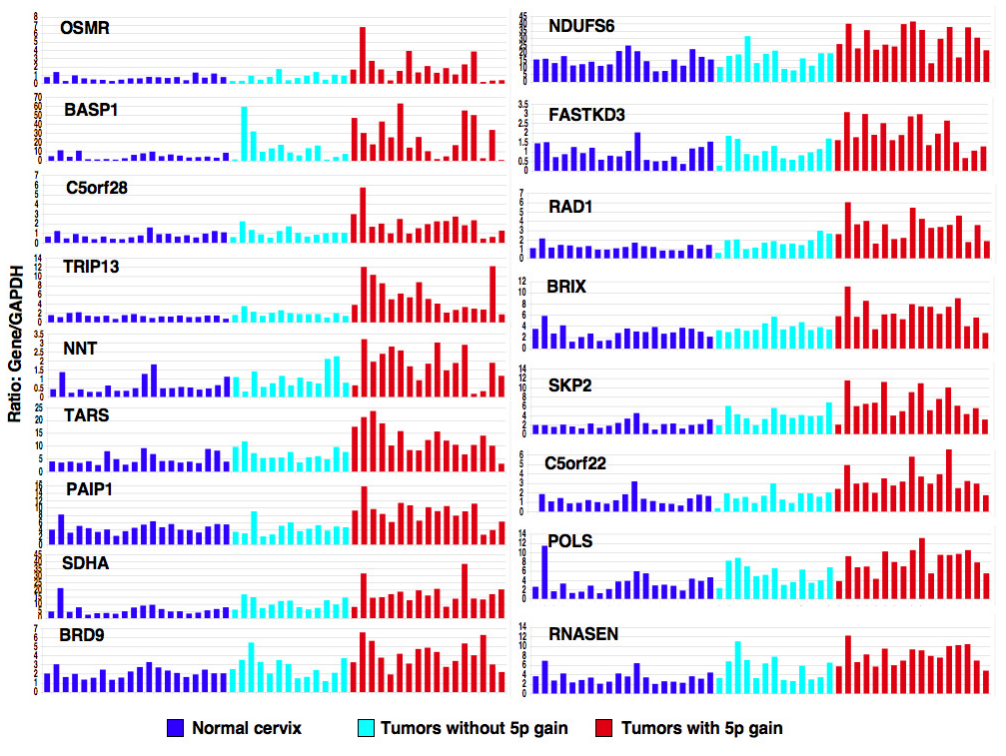
**Relative expression of differentially expressed genes as a consequence of 5p gain in relation to GAPDH in normal and tumors with and without gain of 5p gain**. Genes are shown on top left-side corner of each panel.

Second, we have identified concurrent over-expression (2.2 to 6.0-fold) of genes with 5p gain relative to GAPDH expression (Table 1, Figure 3). Several genes that we found up-regulated in this study as a consequence of 5p gain are potentially relevant to cellular processes associated with tumorigenesis such as signal transduction (*OSMR*), nucleic acid binding, DNA repair, mitotic cycle (*BASP1*, *TARS*, *PAIP1*, *BRD9*, *RAD1*, *SKP2*, and *POLS*), oxidative phosphorylation (*NNT*, *SDHA*, and *NDUFS6*), HPV 16 E1 binding protein (*TRIP13*) [24], ribosomal synthesis (*BXDC2*), micro RNA processing (*RNASEN*).

**Table 1:** Over expressed genes as a consequence of chromosome 5p gain in cervical cancer

**Gene**	**Description**	**Function**	**Role in cancer**	**Average fold change**^#^
*OSMR*	Oncostatin M receptor	Signal transduction	[31]	3.1
*BASP1*	Brain abundant, membrane attached signal protein 1	DNA binding	-	6.0
*C5orf28*	Chromosome 5 open reading frame 28	Not known	-	2.9
*TRIP13*	Thyroid hormone receptor interactor 13	HPV16 E1 protein binding protein	[24]	5.0
*NNT*	Nicotinamide nucleotide transhydrogenase	Detoxification of ROS	-	3.7
*TARS*	Threonyl-tRNA synthetase	Aminoacylation of tRNA	-	3.2
*PAIP1*	Polyadenylate-binding protein-interacting protein 1	Translation activator activity	-	2.3
*SDHA*	Succinate dehydrogenase complex, subunit A, flavoprotein	Oxidative phosphorylation	-	3.4
*NDUFS6*	NADH dehydrogenase (ubiquinone) Fe-S protein 6	Oxidative phosphorylation	-	2.3
*BRD9*	Bromodomain containing 9	Nucleic acid binding	-	2.2
*FASTKD3*	FAST kinase domains 3	Unknown	-	2.4
*RAD1*	RAD1 homolog	DNA repair, cell cycle check point	-	3.2
*BXDC2*	Brix domain containing 2	Ribosomal biosynthesis	-	2.6
*SKP2*	S-phase kinase-associated protein 2	Mitotic check point	[33]	3.6
*C5orf22*	Chromosome 5 open reading frame 22	Unknown	-	3.0
*POLS*	Polymerase (DNA directed) sigma	DNA repair	-	2.8
*RNASEN*	Ribonuclease III, nuclear	miRNA processing	[27]	2.8

# Fold-change is calculated based on comparison between normal cervical squamous epithelium and tumors with Chr5p gain

Of these genes, *RNASEN *(Drosha) over expression was identified in all tumors with 5p gain ascertained by SNP analysis (Figure 2). This finding suggests that *RNASEN *is one of the critical targets conferred by 5p CNI that may play a major role in tumor progression. Drosha executes the initial step in microRNA (miRNA) processing by cleaving pri-miRNA to release pre-miRNA. Drosha is also involved in pre-rRNA processing with specificity to double-strand RNA [25]. Drosha over expression was shown to regulate proliferation and predicts poor prognosis in esophageal cancer [26]. Drosha copy number gain and over expression was shown to influence global miRNA profiles in CC [27]. miRNAs play critical roles in various biological processes including cancer where miRNA fingerprinting can distinguish different lineage tumors [28]. Although the role of Drosha over expression in cancer is not well studied, a number of possibilities exist. Overexpressed Drosha may more efficiently process pri-miRNAs resulting in increased levels of mature miRNAs and the resulting miRNAs may effect transcription of several mRNAs that in turn affect the production of other pri-miRNAs [29]. In the context of its role in miRNA processing, our data suggest that Drosha over expression due to 5p gain is likely an important mechanism in later stages of CC progression.

Previous studies have shown that oncostatin M receptor (*OSMR*) gene is gained and over expressed in CC, which is associated with adverse clinical outcome [30,31]. Oncostatin M (OSM) is a cytokine related to the IL-6 family of cytokines and its biological activity is mediated through the receptor complex. Upon ligand binding, OSMR can activate signaling pathways implicated in cancer such as STAT, PI3/AKT, and mediates inhibition of tumor growth [32]. Angiogenic factor VEGF is induced upon OSM stimulation in cervical cancer cell lines suggesting *OSMR *over expression contributes in CC tumorigenesis [31].

Our expression analysis also showed a number of genes that possess functions related to nucleic acid binding, DNA repair, and mitotic cell cycle (*BASP1*, *TARS*, *PAIP1*, *BRD9*, *RAD1*, *SKP2*, and *POLS*). Of these, the S-phase kinase-associated protein 2 (*SKP2*) plays a critical role in coordinating the G1/S transition, cell cycle progression, forms a substrate recognition subunit of SCF ubiquitin-protein ligase complex, and inhibits the tumor suppressor function of *FOXO1*. Over expression of *SKP2 *was found in many tumor types, consistent with a role of an oncogene, and is associated with poor clinical outcome [33]. *RAD1 *is a component of the 9-1-1 cell-cycle checkpoint response complex that plays a major role in DNA repair [34]. However, its role in cancer is not well understood. Three nuclear genes (*NNT*, *SDHA*, and *NDUFS6*) encoding mitochondrial proteins that play a role in oxidative phosphorylation (OxPhos) were also over expressed as a consequence of 5p gain. The mitochondrial OxPhos system plays a key role in energy production, the generation of free radicals, and apoptosis, the hallmark features of cancer cells [35]. Since tumor cells display enhanced biosynthesis capacity, a key feature of the metabolic transformation of tumor cells that support growth and proliferation, the mitochondrial OxPhos system may stimulate signaling pathways critical in tumor progression. Although nothing is known about these genes in cancer, it remains to be determined whether one or more of these genes act individually or synergistically as oncogenes in regulating the metabolic transformation in CC.

Since genetic activation of therapy targets such as ABL, C-KIT, Her2/neu, and EGFR has been successfully demonstrated to be essential for treatment response [36], our finding of 5p gene targets such as RNASEN, SKP2, and OSMR emphasize the need for functional analysis and dissecting signaling cascades involving these genes in ultimately obtaining therapeutic targets needed for cure and prevention of this devastating cancer.

## Conclusion

In summary, we integrated multiple genomic data to identify 5p gain as the most recurrent chromosomal alteration that occur at high-grade precancerous lesions in the development of CC. We identified the target 5p gain associated over expressed genes that play a role in miRNA processing, signal transduction, DNA repair and mitotic cycle, and oxidative phosphorylation, suggesting a functional role for this chromosomal region in progression of CC. Thus, the genes identified here will form a basis for functional testing of 5p gain and the gene expression levels can be used as a biomarker to identify patients with aggressive disease. Further studies in the context of 5p gain will allow deciphering critical gene targets to develop molecular based therapies for CC.

## Competing interests

The authors declare that they have no competing interests.

## Authors' contributions

LS carried out the SNP analysis, participated in the analysis of SNP and gene expression microarray profile data. GN isolated RNA from tumors, cell lines, generated gene expression profile data and participated in the analysis of SNP and gene expression microarray profile data, SVN and SS generated FISH data, AMF, JDW, BP and AS contributed towards obtaining tumor specimens and collection of clinical information, MM contributed to histological analysis of tumors, generated tissue microarrays and microdissected tumor specimens, HA-F contributed in obtaining tumor specimens, DNA isolation, HPV typing, and clinical information, VVM designed the study, participated in data interpretation and manuscript preparation. All authors read and approved the final manuscript.

## Supplementary Material

Additional file 1 Chromosome summary plot of inferred copy number gain and loss in cervical cancer. Chromosomes marked by solid vertical lines with dotted lines separating short arm (left) and long arm (right). Each SNP value at more than 3 and less than 1 were plotted on horizontal line to show the frequency of gains (above, in red) and losses (below, in blue). Chromosome 5 is highlighted, where 5p gain ranks 1st as the most commonly gained chromosome in cervical cancer. Click here for file

Additional file 2 Clinical and histologic information of CC cases used in SNP, gene expression, and FISH analysis. Specimens used for various types of tests. Click here for file

Additional file 3 Supervised analyses of gene expression profile of chromosome 5 genes identified by comparing and filtering between normal cervix and cervical cancer. In the matrix, each row represents the gene expression relative to group mean and each column represents a sample (shown on Top). T, represents primary tumor; CL, represents cell line. The dendrogram on left shows unsupervised clustering of genes differentially expressed between normal vs. tumor. The dendrogram on top shows unsupervised clustering of tumor and normal. The scale bar (-2 to +2) on the bottom represents the level of expression with intensities of blue represents decrease and red for increase in expression. Click here for file

Additional file 4 Supervised analysis of 26 over expressed genes enriched to identify 5p genes by filtering all over expressed probe sets on chromosome 5 against all 5p probes on U133A array between tumor and normal. In the matrix, each row represents the gene expression relative to group mean and each column represents a sample (shown on Top). T, represents primary tumor; CL, represents cell line. The dendrogram on left shows unsupervised clustering of genes differentially expressed between tumor and normal. The names of genes are shown on right. The scale bar (-2 to +2) on the bottom represents the level of expression with intensities of blue represents decrease and red for increase in expression. The groups within tumors shown at top represent no gain of chromosome 5p (I) and 5p gain (II). Click here for file

Additional file 5 Supervised analysis of 17 genes significantly down regulated on 5q in cervical cancer compared to normal cervix. Down-regulated genes on 5q were identified by filtering all probe sets on chromosome 5q present on U133A array between tumor and normal. In the matrix, each row represents the gene expression relative to group mean and each column represents a sample (shown on Top). T, represents primary tumor; CL, represents cell line. The dendrogram on left shows unsupervised clustering of genes differentially expressed between tumor and normal. The names of genes are shown on right. The scale bar (-2 to +2) on the bottom represents the level of expression with intensities of blue represents decrease and red for increase in expression. The groups within tumors shown at top represent no loss of chromosome 5q (I) and 5q loss (II). Note no significant differences were found between 5q deleted tumors vs. undeleted tumors. Click here for file

## References

[B1] Atkin NB (2000). Significance of chromosome 5 and 17 changes in the development of carcinoma of the cervix uteri. Cytogenet Cell Genet.

[B2] Mitra AB, Rao PH, Pratap M (1994). i(5p) and del(6q) are nonrandom abnormalities in carcinoma cervix uteri. Cytogenetics of two newly developed cell lines. Cancer Genet Cytogenet.

[B3] Rao PH, Arias-Pulido H, Lu XY, Harris CP, Vargas H, Zhang FF, Narayan G, Schneider A, Terry MB, Murty VV (2004). Chromosomal amplifications, 3q gain and deletions of 2q33-q37 are the frequent genetic changes in cervical carcinoma. BMC Cancer.

[B4] Kirchhoff M, Rose H, Petersen BL, Maahr J, Gerdes T, Lundsteen C, Bryndorf T, Kryger-Baggesen N, Christensen L, Engelholm SA, Philip J (1999). Comparative genomic hybridization reveals a recurrent pattern of chromosomal aberrations in severe dysplasia/carcinoma in situ of the cervix and in advanced-stage cervical carcinoma. Genes Chromosomes Cancer.

[B5] Mitra AB, Murty VV, Singh V, Li RG, Pratap M, Sodhani P, Luthra UK, Chaganti RS (1995). Genetic alterations at 5p15: a potential marker for progression of precancerous lesions of the uterine cervix. J Natl Cancer Inst.

[B6] Arias-Pulido H, Narayan G, Vargas H, Mansukhani M, Murty VV (2002). Mapping common deleted regions on 5p15 in cervical carcinoma and their occurrence in precancerous lesions. Mol Cancer.

[B7] Waggoner SE (2003). Cervical cancer. Lancet.

[B8] zur Hausen H (2002). Papillomaviruses and cancer: from basic studies to clinical application. Nat Rev Cancer.

[B9] Gius D, Funk MC, Chuang EY, Feng S, Huettner PC, Nguyen L, Bradbury CM, Mishra M, Gao S, Buttin BM (2007). Profiling microdissected epithelium and stroma to model genomic signatures for cervical carcinogenesis accommodating for covariates. Cancer Res.

[B10] Schwab M (1999). Oncogene amplification in solid tumors. Semin Cancer Biol.

[B11] Pulido HA, Fakruddin MJ, Chatterjee A, Esplin ED, Beleno N, Martinez G, Posso H, Evans GA, Murty VV (2000). Identification of a 6-cM minimal deletion at 11q23.1-23.2 and exclusion of PPP2R1B gene as a deletion target in cervical cancer. Cancer Res.

[B12] Li C, Wong WH (2001). Model-based analysis of oligonucleotide arrays: expression index computation and outlier detection. Proc Natl Acad Sci USA.

[B13] Lin M, Wei LJ, Sellers WR, Lieberfarb M, Wong WH, Li C (2004). dChipSNP: significance curve and clustering of SNP-array-based loss-of-heterozygosity data. Bioinformatics.

[B14] RefGene and Cytoband file. http://biosun1.harvard.edu/complab/dchip/chromosome.htm#refgene.

[B15] Narayan G, Arias-Pulido H, Koul S, Vargas H, Zhang FF, Villella J, Schneider A, Terry MB, Mansukhani M, Murty VV (2003). Frequent Promoter Methylation of CDH1, DAPK, RARB, and HIC1 Genes in Carcinoma of Cervix Uteri: Its Relationship to Clinical Outcome. Mol Cancer.

[B16] Ostor AG (1993). Natural history of cervical intraepithelial neoplasia: a critical review. Int J Gynecol Pathol.

[B17] Murthy NS, Sehgal A, Satyanarayana L, Das DK, Singh V, Das BC, Gupta MM, Mitra AB, Luthra UK (1990). Risk factors related to biological behaviour of precancerous lesions of the uterine cervix. Br J Cancer.

[B18] Schneider A, Koutsky LA (1992). Natural history and epidemiological features of genital HPV infection. IARC Sci Publ.

[B19] Recurrent Chromosome Aberrations in Cancer. http://cgap.nci.nih.gov/Chromosomes/RecurrentAberrations.

[B20] Wilting SM, Snijders PJ, Meijer GA, Ylstra B, Ijssel PR van den, Snijders AM, Albertson DG, Coffa J, Schouten JP, Wiel MA van de (2006). Increased gene copy numbers at chromosome 20q are frequent in both squamous cell carcinomas and adenocarcinomas of the cervix. J Pathol.

[B21] Kloth JN, Oosting J, van Wezel T, Szuhai K, Knijnenburg J, Gorter A, Kenter GG, Fleuren GJ, Jordanova ES (2007). Combined array-comparative genomic hybridization and single-nucleotide polymorphism-loss of heterozygosity analysis reveals complex genetic alterations in cervical cancer. BMC Genomics.

[B22] Heselmeyer K, Macville M, Schrock E, Blegen H, Hellstrom AC, Shah K, Auer G, Ried T (1997). Advanced-stage cervical carcinomas are defined by a recurrent pattern of chromosomal aberrations revealing high genetic instability and a consistent gain of chromosome arm 3q. Genes Chromosomes Cancer.

[B23] Pett MR, Alazawi WO, Roberts I, Dowen S, Smith DI, Stanley MA, Coleman N (2004). Acquisition of high-level chromosomal instability is associated with integration of human papillomavirus type 16 in cervical keratinocytes. Cancer Res.

[B24] Yasugi T, Vidal M, Sakai H, Howley PM, Benson JD (1997). Two classes of human papillomavirus type 16 E1 mutants suggest pleiotropic conformational constraints affecting E1 multimerization, E2 interaction, and interaction with cellular proteins. J Virol.

[B25] Gregory RI, Yan KP, Amuthan G, Chendrimada T, Doratotaj B, Cooch N, Shiekhattar R (2004). The Microprocessor complex mediates the genesis of microRNAs. Nature.

[B26] Sugito N, Ishiguro H, Kuwabara Y, Kimura M, Mitsui A, Kurehara H, Ando T, Mori R, Takashima N, Ogawa R, Fujii Y (2006). RNASEN regulates cell proliferation and affects survival in esophageal cancer patients. Clin Cancer Res.

[B27] Muralidhar B, Goldstein LD, Ng G, Winder DM, Palmer RD, Gooding EL, Barbosa-Morais NL, Mukherjee G, Thorne NP, Roberts I (2007). Global microRNA profiles in cervical squamous cell carcinoma depend on Drosha expression levels. J Pathol.

[B28] Lu J, Getz G, Miska EA, Alvarez-Saavedra E, Lamb J, Peck D, Sweet-Cordero A, Ebert BL, Mak RH, Ferrando AA (2005). MicroRNA expression profiles classify human cancers. Nature.

[B29] Nakamura T, Canaani E, Croce CM (2007). Oncogenic All1 fusion proteins target Drosha-mediated microRNA processing. Proc Natl Acad Sci USA.

[B30] Narayan G, Bourdon V, Chaganti S, Arias-Pulido H, Nandula SV, Rao PH, Gissmann L, Durst M, Schneider A, Pothuri B (2007). Gene dosage alterations revealed by cDNA microarray analysis in cervical cancer: identification of candidate amplified and overexpressed genes. Genes Chromosomes Cancer.

[B31] Ng G, Winder D, Muralidhar B, Gooding E, Roberts I, Pett M, Mukherjee G, Huang J, Coleman N (2007). Gain and overexpression of the oncostatin M receptor occur frequently in cervical squamous cell carcinoma and are associated with adverse clinical outcome. J Pathol.

[B32] Liu J, Hadjokas N, Mosley B, Estrov Z, Spence MJ, Vestal RE (1998). Oncostatin M-specific receptor expression and function in regulating cell proliferation of normal and malignant mammary epithelial cells. Cytokine.

[B33] Gstaiger M, Jordan R, Lim M, Catzavelos C, Mestan J, Slingerland J, Krek W (2001). Skp2 is oncogenic and overexpressed in human cancers. Proc Natl Acad Sci USA.

[B34] Udell CM, Lee SK, Davey S (1998). HRAD1 and MRAD1 encode mammalian homologues of the fission yeast rad1(+) cell cycle checkpoint control gene. Nucleic Acids Res.

[B35] Deberardinis RJ, Sayed N, Ditsworth D, Thompson CB (2008). Brick by brick: metabolism and tumor cell growth. Curr Opin Genet Dev.

[B36] Arteaga CL, Baselga J (2004). Tyrosine kinase inhibitors: why does the current process of clinical development not apply to them?. Cancer Cell.

