# Supplementary figures and images for: Integrative genomics analysis of chromosome 5p gain in cervical cancer reveals target over-expressed genes, including Drosha
Source: Mol Cancer. 2008 Jun 17;7:58. doi: 10.1186/1476-4598-7-58 (PMC2440550; doi:10.1186/1476-4598-7-58)

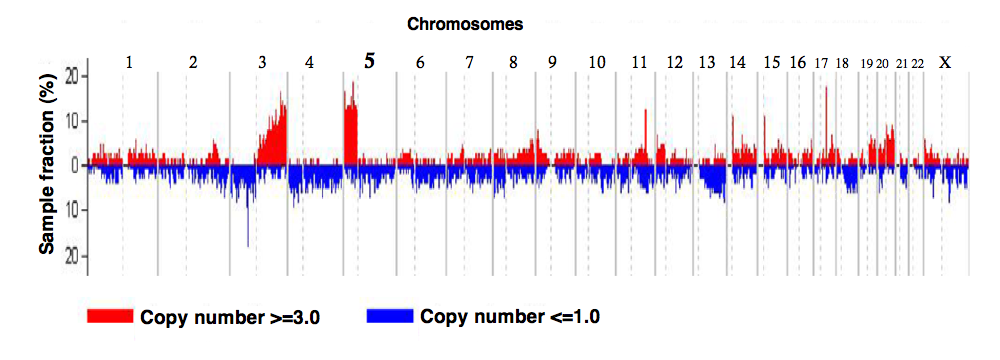

Supplement: Additional file 1 — Chromosome summary plot of inferred copy number gain and loss in cervical cancer. Chromosomes marked by solid vertical lines with dotted lines separating short arm (left) and long arm (right). Each SNP value at more than 3 and less than 1 were plotted on horizontal line to show the frequency of gains (above, in red) and losses (below, in blue). Chromosome 5 is highlighted, where 5p gain ranks 1st as the most commonly gained chromosome in cervical cancer. [file 1476-4598-7-58-S1.tiff]

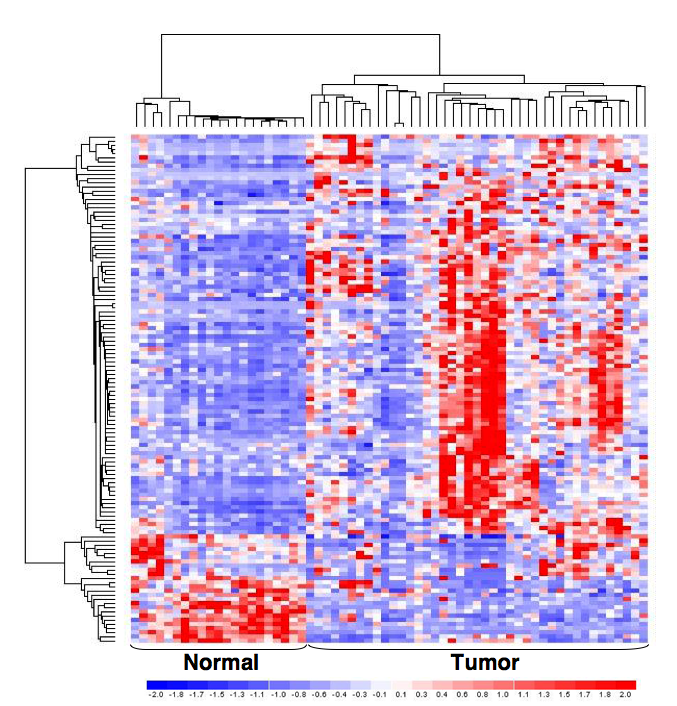

Supplement: Additional file 3 — Supervised analyses of gene expression profile of chromosome 5 genes identified by comparing and filtering between normal cervix and cervical cancer. In the matrix, each row represents the gene expression relative to group mean and each column represents a sample (shown on Top). T, represents primary tumor; CL, represents cell line. The dendrogram on left shows unsupervised clustering of genes differentially expressed between normal vs. tumor. The dendrogram on top shows unsupervised clustering of tumor and normal. The scale bar (-2 to +2) on the bottom represents the level of expression with intensities of blue represents decrease and red for increase in expression. [file 1476-4598-7-58-S3.tiff]

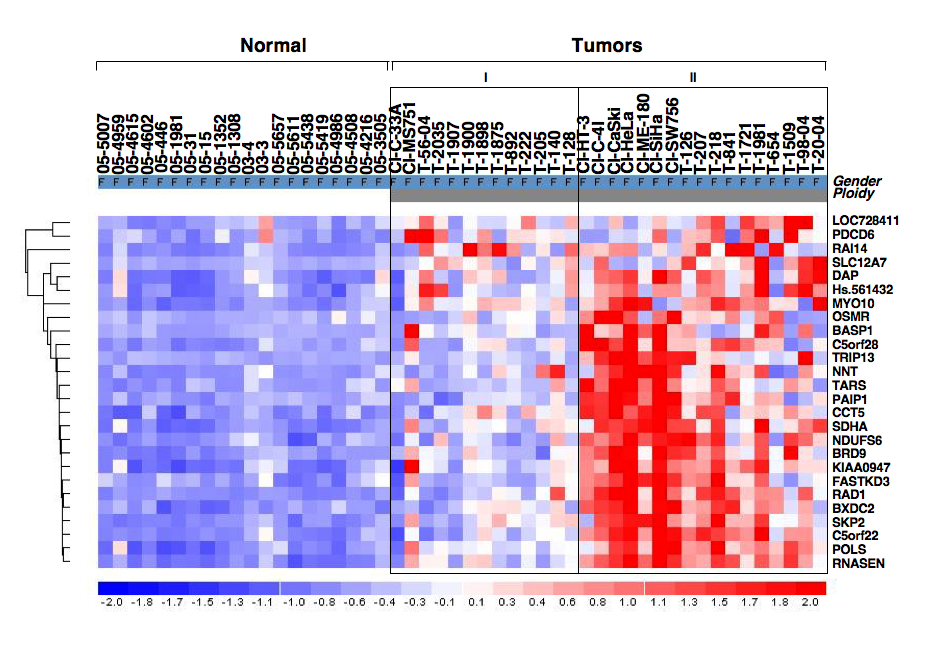

Supplement: Additional file 4 — Supervised analysis of 26 over expressed genes enriched to identify 5p genes by filtering all over expressed probe sets on chromosome 5 against all 5p probes on U133A array between tumor and normal. In the matrix, each row represents the gene expression relative to group mean and each column represents a sample (shown on Top). T, represents primary tumor; CL, represents cell line. The dendrogram on left shows unsupervised clustering of genes differentially expressed between tumor and normal. The names of genes are shown on right. The scale bar (-2 to +2) on the bottom represents the level of expression with intensities of blue represents decrease and red for increase in expression. The groups within tumors shown at top represent no gain of chromosome 5p (I) and 5p gain (II). [file 1476-4598-7-58-S4.tiff]

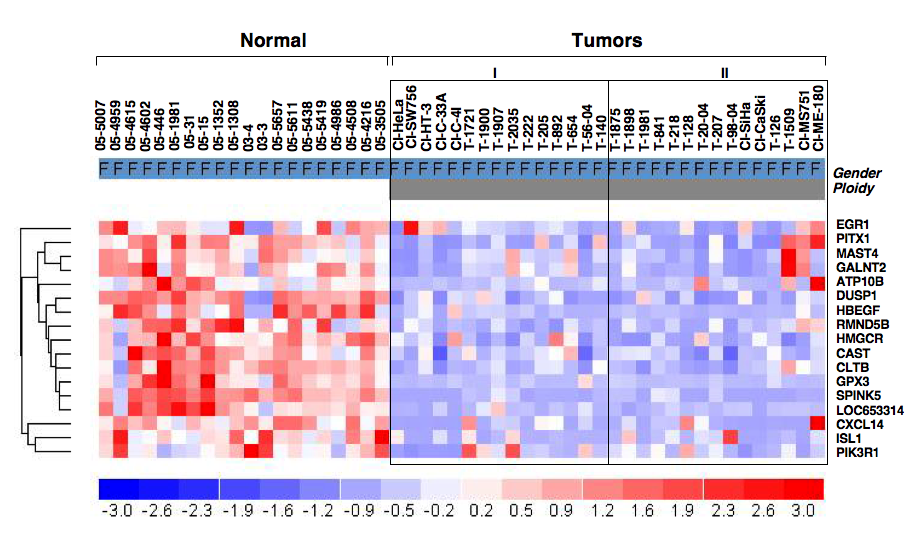

Supplement: Additional file 5 — Supervised analysis of 17 genes significantly down regulated on 5q in cervical cancer compared to normal cervix. Down-regulated genes on 5q were identified by filtering all probe sets on chromosome 5q present on U133A array between tumor and normal. In the matrix, each row represents the gene expression relative to group mean and each column represents a sample (shown on Top). T, represents primary tumor; CL, represents cell line. The dendrogram on left shows unsupervised clustering of genes differentially expressed between tumor and normal. The names of genes are shown on right. The scale bar (-2 to +2) on the bottom represents the level of expression with intensities of blue represents decrease and red for increase in expression. The groups within tumors shown at top represent no loss of chromosome 5q (I) and 5q loss (II). Note no significant differences were found between 5q deleted tumors vs. undeleted tumors. [file 1476-4598-7-58-S5.tiff]
