# Supplementary material for: Integrative genomics analysis of chromosome 5p gain in cervical cancer reveals target over-expressed genes, including Drosha
Source: Mol Cancer. 2008 Jun 17;7:58. doi: 10.1186/1476-4598-7-58 (PMC2440550; doi:10.1186/1476-4598-7-58)
Supplement: Additional file 2 — Clinical and histologic information of CC cases used in SNP, gene expression, and FISH analysis. Specimens used for various types of tests. [file 1476-4598-7-58-S2.doc]

| Additional file 2: Clinical and histologic information of CC cases used in SNP, gene expression, and FISH analysis | | | | | | | | | | | |  |
| --- | --- | --- | --- | --- | --- | --- | --- | --- | --- | --- | --- | --- |
| **S. No.** | **Tumor** | **Histology** | **Stage** | **Size** | **Age** | **Follow up (Months)** | **Treatment** | **Response** | **HPV type** | **SNP** | **U133A** | **5p FISH** |
| 1 | C4I | - | _ | - | - | _ | - | _ | 18 | + | + | ND |
| 2 | Ca Ski | - | _ | - | - | _ | - | _ | 16/18 | + | + | ND |
| 3 | C33-A | - | _ | - | - | _ | - | _ | - | + | + | ND |
| 4 | HT-3 | - | _ | - | - | _ | - | _ | - | + | + | ND |
| 5 | SiHa | SCC | _ | - | - | _ | - | _ | 16 | + | + | ND |
| 6 | SW-756 | SCC | _ | - | - | _ | - | _ | 18 | + | + | ND |
| 7 | MS751 | - | _ | - | - | _ | - | _ | 18/45 | + | + | ND |
| 8 | ME180 | - | _ | - | - | _ | - | _ | 39/68 | + | + | ND |
| 9 | HeLa | AC | - | - | - | - | - | - | 18 | + | + | ND |
| 10 | T-55 | SCC | IIIB | 7 | 33 | 15 | 1 | DOD, PR | 16 | + | ND | ND |
| 11 | T-57 | AC | 1B | 4 | 35 | 29 | 1, 2 | CR, alive | 16 | + | ND | ND |
| 12 | T-66 | SCC | IB2 | 6 | 39 | 23 | 1, 2 | CR, alive | 66 | + | ND | ND |
| 13 | T-75 | SCC | IIIB | 5 | 40 | 39 | 1 | CR, alive | 16 | + | ND | ND |
| 14 | T-78 | SCC | IIIB | 6 | 51 | 10 | 3 | DOD | 16/31 | + | ND | ND |
| 15 | T-79 | SCC | IIIB | 5 | 46 | 21 | 1 | CR | 16 | + | ND | ND |
| 16 | T-80 | SCC | IIB | 8 | 36 | 1 | 3 | DOD | 16 | + | ND | ND |
| 17 | T-86 | SCC | IIB | 3 | 73 | 42 | 2 | CR, alive | 56 | + | ND | ND |
| 18 | T-87 | SCC | 1B2 | - | 27 | 39 | 3 | CR, alive | 45 | + | ND | ND |
| 19 | T-92 | SCC | IIB | 5 | 28 | 2 | 3 | DOD | 67 | + | ND | ND |
| 20 | T-98 | SCC | IIIB | - | 39 | 21 | 1 | DOD, NR | 45 | + | ND | ND |
| 21 | T-103 | SCC | IIB | 6 | 31 | 43 | 1 | CR, alive | 16 | + | ND | ND |
| 22 | T-106 | SCC | IIIB | 10 | 50 | 7 | 1 | DOD, NR | 16 | + | ND | ND |
| 23 | T-107 | SCC | IIIB | 7 | 48 | 8 | 1 | DOD, PR | 16/68 | + | ND | ND |
| 24 | T-108 | SCC | IIIB | 6 | 30 | 32 | 1 | CR | 16 | + | ND | ND |
| 25 | T-114 | SCC | IIB | 4 | 57 | 9 | 2 | DOD, NR | 16 | + | ND | ND |
| 26 | T-116 | SCC | IIIB | 8 | 50 | 5 | 3 | DOD | 16/35 | + | ND | ND |
| 27 | T-117 | SCC | IIIB | 9 | 48 | 2 | 3 | DOD | 16 | + | ND | ND |
| 28 | T-118 | SCC | IIB | 5 | 42 | 12 | 1 | CR, alive | 52 | + | ND | ND |
| 29 | T-124 | SCC | IIIB | 4 | 62 | 36 | 1 | CR, alive | 18 | + | ND | ND |
| 30 | T-126 | SCC | IIIB | 10 | 28 | 6 | 1 | DOD, NR | 45 | + | + | + |
| 31 | T-127 | SCC | IIB | 3 | 65 | 19 | 2 | CR, alive | 16 | + | ND | ND |
| 32 | T-128 | SCC | IIIB | 5 | 55 | 10 | 1 | DOD, NR | 16 | + | + | + |
| 33 | T-130 | SCC | IIA | 1 | 40 | 27 | 2 | CR, alive | 16 | + | ND | ND |
| 34 | T-132 | SCC | 1B1 | 4 | 39 | 23 | 2 | CR, alive | 18 | + | ND | ND |
| 35 | T-133 | SCC | IIIB | 9 | 38 | 2 | 3 | DOD | 16 | + | ND | + |
| 36 | T-134 | SCC | IIIB | 4 | 58 | 13 | 3 | DOD | 39/68 | + | ND | ND |
| 37 | T-135 | SCC | IB1 | 2 | 48 | 33 | 2 | CR, alive | 26/35 | + | ND | ND |
| 38 | T-137 | SCC | IIB | 6 | 42 | 12 | 2 | CR, alive | 16 | + | ND | ND |
| 39 | T-138 | SCC | 1B1 | 1 | 39 | 35 | 2 | CR, alive | 16 | + | ND | ND |
| 40 | T-140 | SCC | IIIB | 5 | 41 | 9 | 1 | DOD, NR | 16 | + | + | + |
| 41 | T-141 | SCC | IIIB | 8 | 37 | 10 | 1 | DOD, PR | 16 | + | ND | + |
| 42 | T-146 | SCC | IIIB | 8 | 51 | 3 | 3 | DOD | 18 | + | ND | + |
| 43 | T-148 | SCC | IIIB | 7 | 60 | 29 | 1 | PR | - | + | ND | ND |
| 44 | T-160 | SCC | IIB | 5 | 39 | 4 | 2 | CR | 39 | + | ND | ND |
| 45 | T-161 | SCC | IB1 | 1 | 39 | 26 | 2 | CR, alive | 16 | + | ND | ND |
| 46 | T-166 | SCC | IIB | 5 | 37 | 15 | 2 | CR | 59 | + | ND | ND |
| 47 | T-167 | SCC | IIIB | 8 | 39 | 24 | 1 | CR | 16 | + | ND | + |
| 48 | T-169 | SCC | IIB | 7 | 34 | 12 | 2 | DOD, NR | 45 | + | ND | ND |
| 49 | T-188 | SCC | IIB | 4 | 77 | 10 | 2 | CR | 58 | + | ND | ND |
| 50 | T-190 | SCC | IIIB | 6 | 28 | 16 | 1 | DOD, PR | 18 | + | ND | ND |
| 51 | T-194 | SCC | IIIB | 6 | 66 | 16 | 1 | CR, alive | 16 | + | ND | ND |
| 52 | T-205 | SCC | IIIB | 7 | 45 | 17 | 1 | DOD | 16 | + | + | + |
| 53 | T-207 | SCC | IIIB | 5 | 54 | 15 | 1 | DOD, NR | 16 | + | + | + |
| 54 | T-218 | SCC | IIIB | 7 | 40 | 15 | 1 | Alive | 16 | + | + | + |
| 55 | T-222 | SCC | IIIB | 10 | 32 | 10 | 2 | DOD | 16 | + | + | + |
| 56 | T-224 | SCC | IIIB | 6 | 38 | 9 | 1 | DOD, NR | 39/6 | + | ND | ND |
| 57 | T-654 | SCC | IA | - | 40 |  | 2 | CR, alive | 45 | + | + | + |
| 58 | T-841 | SCC | IIA | - | 58 |  | 1, 2 | CR, alive | 16 | + | + | + |
| 59 | T-869 | AC | 1B | - | 52 | 72 | 1 | CR | 16 | + | ND | ND |
| 60 | T-892 | SCC | IIIB | - | 34 | 5 | 2- | DOD | 16 | + | + | + |
| 61 | T-939 | AC | IIIB | - | 39 | 5 | 1, 4 | DOD | 18 | + | ND | ND |
| 62 | T-940 | SCC | IA1 | - | 66 | 19 | 1 | DOD | 16 | + | ND | ND |
| 63 | T-954 | SCC | IIB | - | 64 | 56 | 1 | CR | 33 | + | ND | ND |
| 64 | T-966 | SCC | 1B | - | 71 | 57 | 1 | CR | 16 | + | ND | ND |
| 65 | T-968 | AC | IIB | - | 57 | 17 | 1, 4 | DOD | 16/18 | + | ND | ND |
| 66 | T-987 | SCC | 1B | - | 65 | 60 | 2 | CR | 45 | + | ND | ND |
| 67 | T-1051 | SCC | 1B | - | 42 | 60 | 1 | CR | 16 | + | ND | ND |
| 68 | T-1068 | SCC | 1B | - | 41 | 60 | 2 | CR | 16 | + | ND | + |
| 69 | T-1509 | SCC | IIB | - | 69 | NA | 1, 2 | No data | 16 | + | + | + |
| 70 | T-1721 | SCC | IIB | - | 70 | NA | 2 | PR | - | + | + | + |
| 71 | T-1875 | SCC | IIB | - | 36 | NA | 1, 2 | DOD | 16 | + | + | + |
| 72 | T-1898 | SCC | IIB | - | 64 | NA | 2 | DOD | 18 | + | + | + |
| 73 | T-1900 | SCC | IIB | - | 57 | NA | 1, 2 | CR, alive | 18 | + | + | + |
| 74 | T-1907 | SCC | IV | - | 33 | NA | 1, 2 | DOD | 16 | + | + | + |
| 75 | T-1981 | SCC | IB | - | 63 | NA | 1, 2 | DOD | 16/18 | + | + | + |
| 76 | T-2035 | SCC | IIB | - | 59 | NA | 1, 2 | DOD | 18 | + | + | + |
| 77 | T-20-04 | AC | IB2 | - | 46 | NA | NA | NA | ND | + | + | ND |
| 78 | T-56-04 | SCC | IB2 | - | 45 | NA | NA | NA | ND | + | + | ND |
| 79 | T-98-04 | SCC | IIB | - | 38 | NA | NA | NA | ND | + | + | ND |
| Size: in centimeters;  SCC, squamous cell carcinoma; AC, adenocarcinoma  Treatment: 1, radiotherapy; 2, Surgery; 3, no treatment  4, chemotherapy  DOD, died or disease; CR, complete response; PR, partial response  NA, not available' NR, no response; ND, not done | | | | | | | | | | | |  |
